# A phylogenetic group of *Escherichia coli *associated with active left-sided Inflammatory Bowel Disease

**DOI:** 10.1186/1471-2180-9-171

**Published:** 2009-08-20

**Authors:** Andreas M Petersen, Eva M Nielsen, Eva Litrup, Jørn Brynskov, Hengameh Mirsepasi, Karen A Krogfelt

**Affiliations:** 1 Department of Gastroenterology, Hvidovre University Hospital, DK- 2650 Hvidovre, Denmark; 2 Department of Bacteriology, Mycology and Parasitology, Statens Serum Institut, DK-2300 Copenhagen S, Denmark; 3 Department of Gastroenterology, Herlev University Hospital, DK-2730 Herlev, Denmark

## Abstract

**Background:**

*Escherichia coli *have been found in increased numbers in tissues from patients with Inflammatory Bowel Disease (IBD) and adherent-invasive *E. coli *have been found in resected ileum from patients with Crohn's disesae. This study aimed to characterize possible differences in phylogenetic group (triplex PCR), extraintestinal pathogenic *E. coli *(ExPEC) genes and multilocus sequence type (MLST) between *E. coli *strains isolated from IBD patients with past or present involvement of the left side of the colon and from controls.

**Results:**

Fecal samples were collected from 18 patients and from 10 healthy controls. Disease activity was evaluated by sigmoidoscopy. Interestingly, *E. coli *strains of the phylogenetic group B2 were cultured from 60% of patients with IBD compared to 11% of healthy controls (p < 0.05). Furthermore, when comparing the number of *E. coli *B2 strains with at least one positive ExPEC gene among different groups, 86% were found positive among active IBD patients, significantly more than 13% among inactive IBD patients (p < 0.05), and 11% among healthy controls (p < 0.05). The B2 phylogenetic group was found in a specific cluster based on MLST, but no further separation between *E. coli *strains associated with active compared to inactive IBD was achieved.

**Conclusion:**

In conclusion, *E. coli *of the phylogenetic group B2 were isolated more frequently from IBD patients with past or present involvement of the left side of the colon compared to healthy controls, and B2 strains with ExPEC genes were found more frequently among IBD patients with active disease compared to patients with inactive disease.

## Background

The pathogenic mechanisms of inflammatory bowel disease (IBD) have been researched intensely. In general, it is believed that both genetic and environmental factors are involved. When IBD was originally described, a close resemblance to infectious diseases of the gut was noticed. Therefore, many different bacteria, viruses and other microorganisms have been suspected to cause IBD. It is now well established that luminal factors in the intestine are involved in the inflammatory process of Crohn's disease (CD) and ulcerative colitis (UC). For example, diversion of the continuity of the intestines results in healing of the resting gut, whereas the inflammation will return when continuity is reestablished [1]. Furthermore, several animal models have documented the participation of bacteria in the inflammatory process [2]. More importantly, the recent finding of a defect in the caspase recruitment domain family, member 15 (NOD2/CARD15), gene among CD patients, has reawakened the search for specific involved pathogens [3]. NOD2/CARD15 is believed to be involved in the innate immune system including the production of defensins; therefore, defects in this gene could indicate that the host is more susceptible to microorganisms [4]. It has also been shown that the number of viable internalized *S. typhimurium *in Caco2 cells was higher when the Caco2 cells were transfected with a variant CARD15/NOD2 expression plasmid associated with Crohn's disease [5].

*Escherichia coli *are among the most interesting bacteria in the human gut. Certain *E. coli *clones with specific virulence factors are involved in extraintestinal infections the so called extraintestinal pathoghenic *E. coli *(ExPEC), and these bacteria often cause both urinary tract infections and septicemia. Furthermore, specific *E. coli *are involved in childhood diarrhea, (enteropathogenic *E. coli*), tourist diarrhea (enterotoxigenic *E. coli*), and recently, bloody diarrhea associated with hemolytic uremic syndrome (verotoxin-producing *E. coli*).

In the 1970's, it was found that hemolytic *E. coli *were linked to active UC, although it was believed that the hemolytic *E. coli *were innocent bystanders, and their presence in the colon was assisted by the inflammation but did not cause it [6]. On the other hand, it has been shown that apathogenic *E. coli *prevents relapse of UC just as well as mesalazine [7]. Furthermore, *E. coli *has been linked to CD, since an abundance of specific adherent-invasive *E. coli *was found in resected ileum from patients with CD, compared to non inflamed ileum resected due to other causes [8,9]. Very recently, it was demonstrated by ribosomal intergenic spacer analysis that enterobacteriaceae are more abundant in tissue samples from patients with IBD compared to controls, and after culture, specific phylogenetic groups of *E. coli *were found to be more frequent among patients with UC and CD [10]. Moreover, it has been shown that *E. coli *are very predominant in inflamed mucosa of patients with UC, and that these strains based on 16 S rRNA PCR are "active" and overrepresented in comparison with the microbiota of healthy controls, who generally had a higher biodiversity of the active microbiota [11]. In addition, an exuberant inflammatory response to *E. coli *has been demonstrated among patients with UC [12].

The aim of our study was to characterize possible differences in phylogenetic group, serotype, ExPEC genes and virulence between *E. coli *isolated from patients with active IBD, patients with inactive disease and healthy controls, as well as to examine whether multilocus sequence typing (MLST) could further distinguish between these *E. coli*. MLST is considered the most stable and appropriate of currently available molecular typing techniques for long term epidemiology and for the identification of bacterial lineages that have an increased propensity to cause disease [13].

## Results

Fecal samples were collected from 18 patients with IBD with present or past involvement of the left side of the colon and from 10 healthy controls. In both patients and controls, a sigmoidoscopy was performed. Ten patients were found to have a non-inflamed mucosa, whereas 8 had clear inflammation in the sigmoid colon. More detailed characteristics of patients are presented in Table 1. A total of 26 *E. coli *strains were isolated from study subjects. From 3 patients and 1 control, no *E. coli *could be isolated. From one patient with active IBD and one patient with inactive IBD two different *E. coli *strains from each patient were isolated based on MLST, serotyping and phylogenetic typing. From all controls and all other patients only one strain of *E. coli *from each subject was isolated.

**Table 1:** Characteristics of patients with active and inactive inflammatory bowel disease (IBD) and of controls.

	Controls	Inactive UC	Active UC	Inactive CD	Active CD
N	10	5	6	5	2

id numbers	c^1^1, c2, c3, c4, c5, c6, c12, c14, c16, c17	p10, p23, p26, p27, p32	p7, p8, p13, p19, p22, p25	p11, p15, p18, p20, p31	p29, p30

M/F	6/4	2/13	5/1	1/4	2/0

mean age	27 (21–33)	40 (37–54)	42 (34–71)	48 (34–65)	48

localization of disease, (present when active, previous when inactive)	None	Proctosigmoid colon (p10, p23, p26), pancolitis (p32), rectum (p27)	rectum (p8), proctosigmoid colon (p7, p19, p22), pancolitis (p13, p25)	descending colon (p15, p18, p20), proctosigmoid colon (p14, p31)	colon with skip lesions (p29), proctosigmoid colon (p30)

Medication	None	5-ASA (p10, p23, p26), azathioprine (p27), none (p32)	5-ASA (all), Azathioprine (p13, p19), prednisolone (p13)	None (p15, p18, p31), 5-ASA (p20), prednisolone (p11)	5-ASA (p29), none (p30)

UC; Ulcerative Colits, CD; Crohn's disease.

Controls have the prefix "c" and patients "p".

**Figure 1:**
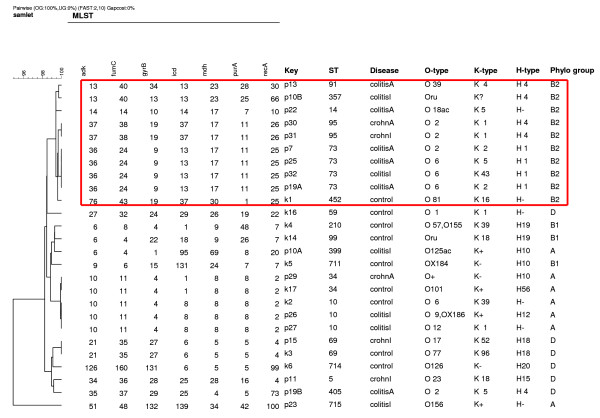
**Phylogenetic tree based on Multilocus Sequence Typing of *Escherichia coli *isolated from fecal samples from patients with active and inactive Inflammatory Bowel Disease, all with past or present involvement of the left side of the colon, and from controls**. Also presented is serotype and phylogenetic groups A, B1, B2 or D. B2 strains are marked with a red box. ColitisI; inactive Ulcerative Colitis, colitisA; active Ulcerative Colitis, crohnI, inactive Crohn's disease, crohnA; active Crohn's disease. ST; sequence type.

*E. coli *strains were studied with respect to phylogenetic group, ExPEC genes, multilocus sequence type, serotype and virulence factors. Interestingly, among patients and controls with a positive *E. coli *culture, B2 strains were cultured most frequently from patients with IBD, 60% (9 out of 15), compared to 11% (1 out of 9) from healthy controls (p < 0.05). In addition, B2 *E. coli *strains were cultured most frequently from patients with active IBD, 86% (6 of 7), compared to 38% (3 of 8) among patients with inactive colitis, but this difference did not reach statistical significance (p = 0.12). However, when comparing the number of B2 *E. coli *strains with at least one positive ExPEC gene among different groups (table 2), significantly more strains, 86% (6 of 7), were found positive among active IBD patients, compared to 13% (1 of 8) among inactive IBD patients (p < 0.05) and 11% (1 of 9) among healthy controls (p < 0.05). Among the 26 *E. coli *strains, representing 20 O-serogroups, 18 sequence types were identified using multilocus sequence typing (MLST) (figure 1). The B2 phylogenetic group associated with IBD was found in a specific cluster based on MLST, confirming a common ancestry of these IBD associated B2 *E. coli*, but no further separation was achieved between strains involved in active compared to inactive IBD. From most patients with active IBD, 71%, *E. coli *were cultured with O-serotypes normally categorized as uropathogenic, compared to 25% (p = 0.13) in IBD in remission and 11% among healthy controls (p < 0.05). Although hemolytic *E. coli *were isolated more frequently from patients with IBD (47%) compared to healthy controls (11%); this difference did not reach statistical significance (p = 0.18).

**Table 2:** ExPEC genes in *Escherichia coli *isolated from fecal samples from patients with active and inactive IBD and from controls.

Disease-Group	Reference number	*Pap A *717 bp	*afa *594 bp	*Sfa/foc *410 bp	*Iut *302 bp	*kpsM II *272 bp	*Pap C *205 bp	phylogenetic group
**control**	**c1**	**-**	**-**	**+**	**-**	**+**	**-**	**B2**
control	c2	-	-	+	-	-	-	A
control	c3	-	-	-	-	-	-	D
control	c4	-	-	-	+	-	-	B1
control	c5	-	-	-	-	-	-	B1
control	c6	-	-	-	-	-	-	D
control	c14	-	-	-	-	-	-	B1
control	c16	+	-	-	-	+	-	D
control	c17	-	-	-	-	-	-	A

IBDI	p10A	-	-	-	-	-	-	A
	p10B	-	-	-	-	-	-	B2
IBDI	p11	+	-	-	-	+	-	D
IBDI	p15	-	+	-	+	+	+	D
IBDI	p23	-	-	-	-	-	-	A
IBDI	p26	-	-	-	-	-	-	A
IBDI	p27	-	+	-	-	+	-	A
IBDI	p31	-	-	-	-	-	-	B2
**IBDI**	**p32**	**-**	**-**	**+**	**+**	**-**	**-**	**B2**

**IBDA**	**p7**	**+**	**+**	**+**	**+**	**-**	**+**	**B2**
**IBDA**	**p13**	**-**	**-**	**-**	**-**	**+**	**-**	**B2**
**IBDA**	**p19A**	**-**	**+**	**+**	**+**	**-**	**+**	**B2**
	p19B	-	+	-	-	+	-	D
**IBDA**	**p22**	**+**	**-**	**+**	**+**	**+**	**+**	**B2**
**IBDA**	**p25**	**+**	**-**	**+**	**+**	**+**	**+**	**B2**
IBDA	p29	-	-	-	-	-	-	A
**IBDA**	**p30**	**-**	**-**	**-**	**+**	**-**	**+**	**B2**

B2 strains with at least one positive ExPEC gene in bold.

No verotoxin producing strains were detected among the 26 *E. coli *isolates examined, and no other common virulence genes were significantly associated with disease activity based on hybridization assays (table 3).

**Table 3:** Serotype and phenotype of *Escherichia coli *isolated from fecal samples from patients with active and inactive IBD and from controls.

Disease group	Reference number	Virulence Genes	O TYPE	K TYPE	H TYPE	Hemolysin
Control	c1	-	O81	K16	H-	-
Control	c2	*astA*	**O6**	K39	H-	-
Control	c3	-	O77	K96	H18	-
Control	c4	-	O57, O155	K39	H19	-
Control	c5	-	OX184	K-	H10	-
Control	c6	-	O126	K-	H20	-
Control	c12	ND				
Control	c14	-	Oru	K18	H19	-
Control	c16	-	O1	K1	H-	Ent
Control	c17	*astA*	O101	K+	H56	-

IBD Inactive	p10A	-	O125ac	K+	H10	-
	p10B	-	Oru	K?	H4	-
IBD Inactive	p11	-	O23	K18	H15	Ent.
IBD Inactive	p15	*astA*	O17	K52	H18	**-**
IBD Inactive	p18	ND				
IBD Inactive	p20	ND				
IBD Inactive	p23	-	O156	K+	H-	-
IBD Inactive	p26	-	O9, OX186	K+	H12	Ent.
IBD Inactive	p27	-	O12	K1	H-	Ent.
IBD inactive	p31	-	**O2**	K1	H4	-
IBD Inactive	p32		**O6**	K43	H1	-

IBD Active	p7	*astA*	**O2**	K2	H1	Ent
IBD Active	p8	ND				
IBD Active	p13	-	O39	K4	H4	-
IBD Active	p19A	-	**O6**	K2	H1	Alpha
	p19B	*SLM862*	**O2**	K5	H4	-
IBD Active	p22	-	**O18ac**	K5	H-	Alpha
IBD Active	p25	*astA*	**O6**	K5	H1	Ent.
IBD Active	p29	*aatA*	O+	K-	H10	-
IBD Active	p30	-	**O2**	K1	H4	-

Uropathogenic E. coli associated O-type in bold.

## Discussion

In our study based on fecal samples from patients with previous or present left-sided colitis and from controls, we found a strong correlation between isolation of *E. coli *of the phylogenetic group B2 and IBD; no correlation was found with other phylogenetic groups including group D. Further, we found a trend toward an association between the presence of B2 *E. coli *and active colitis. A recent study has demonstrated that the presence of specific *E. coli *(both groups B2 and D), in colonic biopsies, are associated with IBD, however patients were not stratified according to activity of the disease or to disease localization [10]. Our patients were well-defined regarding disease localization (left-sided colitis), which could explain the very specific association between B2 *E. coli *and IBD in our study. Controls (medical students) were younger than IBD patients, however, in broad terms the colonic microbiota is generally viewed as being a stable entity within an individual [14]. Moreover, previous studies of B2 *E. coli *did not show an increase in the probability of detecting a B2 *E. coli *with increasing age in the age groups participating in our study [15].

B2 strains are often found among ExPEC strains and when testing for 6 genes commonly associated with ExPEC [16], we found a statistically significant association between active IBD and B2 strains with at least one positive ExPEC gene, when comparing to both controls and to patients with inactive disease. The enhanced virulence potential of ExPEC strains is thought to be caused mainly by their multiple virulence factors such as adhesins, siderophores, toxin polysaccharide coatings; e.g., these virulence factors would help the bacteria to avoid host defenses, injure or invade host cells and tissues and stimulate a noxious inflammatory response [17]. It has been suggested that features, which commonly have been regarded as virulence factors in ExPEC isolates, are also factors promoting intestinal colonization [18-20]. This could explain why ExPEC strains are more prevalent in patients with UC, where the inflamed mucosa could prevent colonization with *E. coli *of a more commensal nature.

Whether IBD associated B2 *E. coli *can be differentiated from other B2 ExPEC strains is at present not known. In this regard it was interesting to find a possible association of the IBD associated B2 *E. coli *with *afa*, afimbrial adhesin, an adhesin which exist in different subtypes depending on the physiological site from which the *afa *positive *E. coli *were isolated [21]. Furthermore, the afimbrial adhesin has been demonstrated to cause functional lesions in the intestinal brush border, impairment of the epithelial barrier and proinflammatory responses in cultured human intestinal cells that express the structural and functional characteristics of human enterocytes [22].

MLST confirmed the common ancestry of the B2 *E. coli*, since they were all found in the same phylogenetic group, but unfortunately, no further information could be obtained regarding stratification of the B2 *E. coli *from active IBD patients compared to inactive IBD patients. Previously B2 *E. coli *strains have been described using MLST and subgroups have been identified [23], but our B2 strains were of sequence types typically found in different subgroups, making further information regarding these strains using MLST unlikely.

## Conclusion

In conclusion, the clonal nature, based on MLST and phylogenetic group, of *E. coli *isolates from IBD patients with left-sided colitis contradicts an assumption that IBD through an impaired immune system simply allows an overrepresentation of *E. coli *at random. Some active participation by the microorganism is certainly indicated, either due to colonization advantages or as a part of IBD pathogenesis. Future studies of the effects of IBD associated *E. coli *in both cell assays and animal models will help to clarify the role of these bacteria in the inflammatory process.

## Methods

### Subjects

Permission for the study was obtained from the Regional Ethics Committee for Copenhagen County Hospitals (Permission no. KA03019) and all participants gave their informed written consent. Controls were recruited among medical students. All controls had a completely normal distal colon as visualized by video sigmoidoscopy at study entry. Patients with IBD were diagnosed according to standardised criteria [24,25], which included a fresh set of negative stool cultures for common pathogens including *Clostridium difficile*. All patients with CD had previous or present involvement of the left side of the colon. The basic clinical features of the study groups are presented in Table 1

### Samples and selection of *E. coli *isolates

Fecal samples from patients and controls were used in this study. Fecal samples were collected by patients and controls and submitted for culture at the Department of Bacteriology, Mycology and Parasitology, Statens Serum Institut, Copenhagen, Denmark, and *E. coli *colonies were chosen for further characterization by a lab technician without knowledge of the clinical data of the participating patients and controls.

### Microbiological methods

Fecal cultures were performed by suspending 10 μl or an amount equivalent to 10 μl feces into 2 ml of phosphate-buffered saline (pH 7.38). The suspension was mixed, and 10 μl was plated on SSI enteric medium [26] and incubated at 37°C overnight. The plates were examined for the colony characteristics, size, and colour of the cultured organisms. Colonies with characteristic features for *E. coli *were chosen for colony blot hybridization, serotyping and MLST. The strains were confirmed as being *E. coli *by using the Minibact E kit (Statens Serum Institut, Copenhagen, Denmark) [27]

### Serotyping

The isolates were serotyped according to standard methods [28] using the full set of antisera (Statens Serum Institut, Hillerød, Denmark).

### DNA hybridization

Virulence genes of common *E. coli *pathotypes were detected by DNA probe-hybridisation assays: verocytotoxin genes (*vtx1, vtx2*) intimin (*eae*), enterohemolysin (*ehxA*), bundle-forming pili *(bfpA)*, EAST1 (*astA*), marker for enteroaggregative *E. coli *(*aatA/*CVD432) and marker for diffuse adherence (SLM862) as described previously [29,30].

### MLST

MLST was performed according to the scheme described at the *E. coli *MLST website maintained at the Max-Planck Institut für Infektionsbiologie http://web.mpiib-berlin.mpg.de. The seven housekeeping genes were shown to be unlinked on an *E. coli *K-12 genome map. Product lengths varied from 583 to 932 bp. DNA was isolated from the colonies using the ChargeSwitch^® ^gDNA Mini Bacteria Kit (Invitrogen, Carlsbad, CA, USA), and stored at -20°C until required for PCR amplification.

### Sequencing

PCR reactions were performed on the purified DNA using PuReTaq Ready-To-Go™ PCR beads (Amersham Biosciences UK Limited, Buckinghamshire, England) by adding 1 μl of extracted DNA (~10 ng DNA), 1 μl of each primer (10 pmol μl^-1^) and 22 μl of water Mini-plasco^® ^(Braun Melsungen AG, Melsungen, Germany). Primer sequences and cycling conditions were employed as described on the MLST website. PCR was performed on a GeneAmp^® ^PCR System 9700 (Applied Biosystems, Foster City, CA, USA). PCR products were purified with the ChargeSwitch^® ^PCR Clean-Up Kit (Invitrogen) and sequenced by MWG Biotech (Ebersberg, Germany).

### Sequence analysis

Raw sequences were reviewed by visual inspection in BioNumerics version 4.601 (Applied Maths, Sint-Martens-Latem, Beligium). DNA sequences were aligned and trimmed. Obtained sequences were aligned against known alleles in the database at the website, and allele numbers and sequence types were assigned. In the case of unknown alleles and/or sequence types, the new alleles and sequence types were submitted to the database. The phylogenetic tree is an UPGMA tree calculated in BioNumerics on the basis of the concatenated sequences.

### Phylogenetic group

Phylogenetic groups (A, B1, B2 and D) were determined by a simple PCR procedure based on genes *chuA*, *YjaA *and an anonymous DNA fragment, using primers and conditions exactly as described by Clermont et al [31].

### ExPEC genes

The presence of six ExPEC genes, *papA *(P fimbriae), *papC *(pilus assembly), *afa *(afimbrial adhesion), *sfa/foc *(Sfimbriae/F1Ccfimbriae), *iut *(aerobactin system) and *kpsM *(kapsular synthesis) was detected by a PCR method, using primers and conditions exactly as described by Johnson et al [16].

### Statistics

The number of hemolysin positive *E. coli*, *E. coli *of serotypes typical for ExPEC, *E. coli*, with at least one positive ExPEC gene and B2 *E. coli *in different clinical groups were assessed with the Fisher Exact test (2-tailed). P < 0.05 was considered significant.

## Authors' contributions

AMP, JB, KAK participated in the design of the study. AMP and JB contacted patients and controls and performed the sigmoidoscopies, KAK was responsible for isolation of *E. coli *and microbiological tests. AMP and KAK drafted the manuscript and performed the statistical analysis. EMN, EVL and HMI performed the molecular genetic studies and serotyping. All authors read and approved the final manuscript.
